# Changes in the electromyographic activity of masticatory muscles in patients undergoing bimaxillary surgery

**DOI:** 10.2340/aos.v84.43408

**Published:** 2025-04-22

**Authors:** Ali Kağan Özen, İsmail Ceylan

**Affiliations:** a Department of Orthodontics, Kafkas University Faculty of Dentistry, Kars, Türkiye; b Department of Orthodontics, Atatürk University Faculty of Dentistry, Erzurum, Türkiye

**Keywords:** Masticatory muscles, electromyography, orthognathic surgery, muscle contraction

## Abstract

**Objective:**

The aim of this study is to investigate short-term changes in the electromyographic (EMG) activity of masticatory muscles in individuals with skeletal Class III malocclusion undergo double-jaw orthognathic surgery.

**Material and methods:**

In patients with skeletal Class III anomaly, EMG activity changes in the anterior temporalis and masseter muscles were measured before T0 and at 3 (T1) and 6 (T2) months after bimaxillary orthognathic surgery. Recordings were obtained using the ‘MP100’ device and circular Ag-AgCl electrodes during closure, clenching, chewing and swallowing. Data from 26 individuals (12 males, 14 females) with a mean age of 21.7 years were analysed using the Friedman test.

**Results:**

A significant decrease was observed in the right/left masseter muscles during clenching/chewing from T1 to T0 (right and left masseter clenching: *p* < 0.001, left masseter chewing: *p* < 0.01, right masseter chewing: *p* < 0.05), while a significant increase was noted in the right masseter’s clenching function from T2 to T1 (*p* < 0.01). The EMG activity of the right anterior temporal muscle decreased during clenching at T1–T0 (*p* < 0.001), increased at T2–T1 (*p* < 0.05), and decreased during chewing/swallowing at T1–T0 (chewing: *p* < 0.001, swallowing: *p* < 0.05) and T2–T0 (*p* < 0.05). The left anterior temporal muscle showed decreased EMG activity during clenching at T1–T0 (*p* < 0.001), increased at T2–T1 (*p* < 0.05), and decreased during chewing at T1–T0 (*p* < 0.001). During swallowing, a decrease was observed at T2–T0 (*p* < 0.001).

**Conclusions:**

Partial changes in EMG activity were observed during some functions in the 3-month period; however, no significant overall change was recorded in the 6-month period.

## Introduction

Class III malocclusions, while among the least prevalent, can present in various clinical forms, including retrognathic maxilla, prognathic mandible, or a combination of both [1]. It is known that individuals with this anomaly have a greater need for orthognathic surgery compared to those with other anomalies, such as Class II malocclusion, due to functional and aesthetic problems [2].

While dental and skeletal anomalies are treated through orthodontic and orthognathic approaches, it is an inevitable fact that the soft tissues anatomically adjacent to these structures are also affected by these treatments. In other words, it should be remembered that there is a mutual interaction between anomalies in soft and/or hard tissues [3, 4].

The masticatory muscles, one of the affected soft tissues, are a group of muscles that work in an organised manner with each other and the lower jaw joint during mandibular movements. This group, consisting of the temporalis, masseter, lateral and medial pterygoid muscles, generally extends between the cranial skeleton and the mandible [5–7]. Electromyography is one of the methods of evaluation in the activity of masticatory muscles [8]. It is the process of measuring the electrical potentials that arise from the combined contractions of multiple muscle fibres because of the action potential occurring in a muscle fibre, recorded through electrodes to assess the activity of nerves and muscle fibres [9]. This process provides information about the status of the motor nerve exiting from the anterior horn of the spinal cord and the muscle fibre group it stimulates [10].

Due to the mutual interaction between soft and hard structures, a wide range of electromyographic (EMG) assessments have been conducted, from the degree to which muscle activities are affected by anomalies in orthodontics to the muscle responses that arise as a result of orthodontic treatment [11–19]. Studies have been conducted on patients with skeletal deformities to examine the effects of existing deformities on muscles and to evaluate the significant changes brought about by orthognathic surgical approaches [20–28].

Di Palma et al. evaluated the EMG activities of the anterior temporal and masseter muscles in patients with skeletal Class II who underwent mandibular surgery and those with skeletal Class III who underwent bimaxillary surgery. The researchers reported that after the operation, there was a better balance between the right and left muscles in terms of EMG data, although not significantly, and the effect of lateral deviation in the mandible decreased [20].

Frongia et al. assessed the changes in EMG values of the masseter and anterior temporal muscles in patients with skeletal Class III after single or bimaxillary surgery based on ‘activity’, ‘asymmetry’, and ‘torque’ indices. They stated that after orthognathic surgery, the anterior temporal muscle became dominant over the masseter muscle during activity, and both asymmetry and torque index values for the right and left muscles regressed towards the normal range [22].

Ko et al. evaluated the EMG activities of the anterior temporal and masseter muscles in individuals with skeletal Class III anomalies following double-jaw surgery. The researchers reported a significant decrease in activity in both muscles 1 month after the surgery, followed by an observed increase in muscle activity. However, in the long term, they noted that the muscle activities approached pre-operative values [23].

Muftuoglu et al. assessed the changes in the EMG activity of the masseter muscle after bimaxillary surgery in patients with skeletal Class III, comparing both periods and the control group. The researchers reported that there was no significant difference in EMG data at rest, while the EMG values for maximum voluntary clenching significantly increased after surgery, although they remained lower than those of the control group across all periods [29].

In the literature examining the relationships between orthognathic surgery and EMG activity of masticatory muscles, no study has been found that simultaneously evaluates closing, clenching, chewing, and swallowing functions. In addition, in the context of orthognathic surgery, studies that assess chewing function using a real food item like nuts are also quite scarce [30–33].

Moreover, some studies do not standardise the type of surgery performed (e.g. only maxillary, only mandibular, or bimaxillary groups), which is also noteworthy [24, 29, 34, 35].

Due to these observations in the literature, we considered it more original to conduct our study exclusively on patients undergoing bimaxillary surgery and to evaluate all activities – closing, clenching, chewing (with nuts) and swallowing – together.

The present study aimed to investigate the short-term changes in EMG activity of masticatory muscles in individuals with skeletal class III malocclusion who underwent double jaw orthognathic surgery. Our null hypothesis (H₀) is as follows: ‘Bimaxillary orthognathic surgery does not cause a significant change in the EMG activity of the masticatory muscles’.

## Methodology

The ethical compliance of this study was unanimously approved by the ‘Clinical Research Ethics Committee of Atatürk University Medical Faculty’ on December 30, 2021, with the decision number B.30.2.ATA.0.01.00/28. The volunteers were informed about the study, provided with details regarding the protection of their rights, and signed informed consent forms were obtained.

In our study, for EMG assessments, we based our methodology on the study by Çelakıl et al., titled ‘Effect of orthognathic surgery on masticatory performance and muscle activity in skeletal Class III patients’ [21]. According to a G* Power analysis, to observe a statistically significant difference in EMG activity of the masseter muscle during maximum bite force between T3 (96.22 ± 13.27 μV) and T0 (70.07 ± 15.74 μV) at 80% power and 95% confidence intervals (CIs), a sample size of 20 patients is required.

Our inclusion criteria required patients to have a skeletal Class III relationship severe enough to necessitate bimaxillary surgery and to have completed their pubertal growth spurt. Our exclusion criteria included a history of previous orthodontic treatment, temporomandibular joint disorders, congenital and/or genetic craniofacial or cervical anomalies (such as cleft lip and/or palate), any muscular or neurological disorders, a history of condylar fracture, or facial trauma.

Our study was conducted on the EMG recordings of 26 patients who applied for treatment at the Atatürk University Faculty of Dentistry, Department of Orthodontics, and underwent double-jaw surgery (maxillary advancement and mandibular setback). Electromyographic recordings were taken 1 week before the operation (T0), and at 3 months (T1) and 6 months (T2) postoperatively. All operations were performed by the same surgeon.

**Figure 1:**
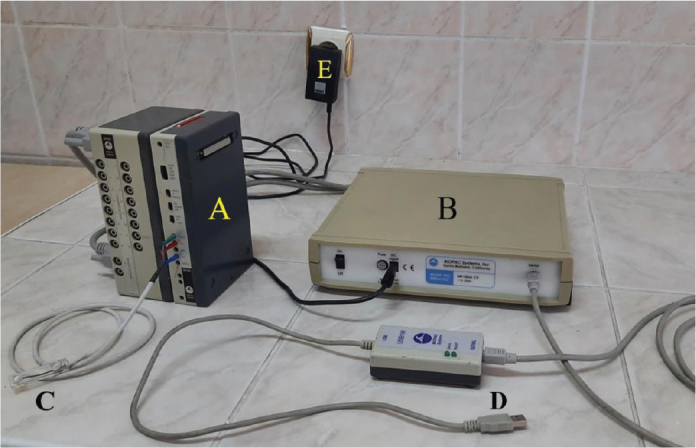
(A) Electromyogram amplifier module. (B) Data acquisition unit. (C) Surface Ag-AgCl electrodes. (D) USB converter. (E) Power converter.

All EMG recordings were obtained using the ‘MP 100’ device (Biopac System Inc, 42 Aero Camino, Goleta, CA, 93117, USA) (Figure 1) located within the Department of Physiology at Atatürk University Medical Faculty, and the recordings were evaluated using the ‘AcqKnowledge’ software (version 3.9.1) produced by the same company.

**Figure 2:**
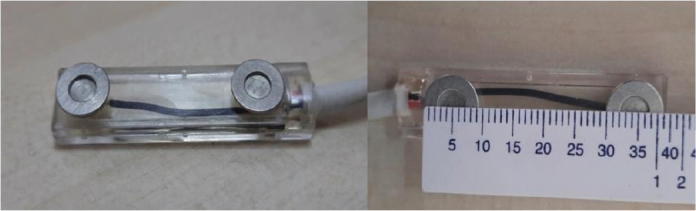
Surface electrodes.

Before each recording, the skin area to be recorded was cleaned with an alcohol swab. To remove dead cells from the skin and reduce electrical resistance, an abrasive paste (Nuprep^®^ Skin Prep Gel, Weaver and Company, USA) was applied using a gauze pad in circular motions, and the remnants of this paste were cleaned afterward. To enhance electrical conductivity, a conductive cream (AFC EEG Conductive Paste, AFC Medical, TÜRKİYE) was applied to the relevant area using cotton. After cleaning the measurement area with alcohol, applying the abrasive paste and conductive cream, the skin was allowed to absorb the conductive cream for 5 min before starting the EMG recordings [36, 37]. The circular surface electrodes with a diameter of 9 mm were used during the recordings, with a distance of 30 mm between their centres (Figure 2).

**Figure 3:**
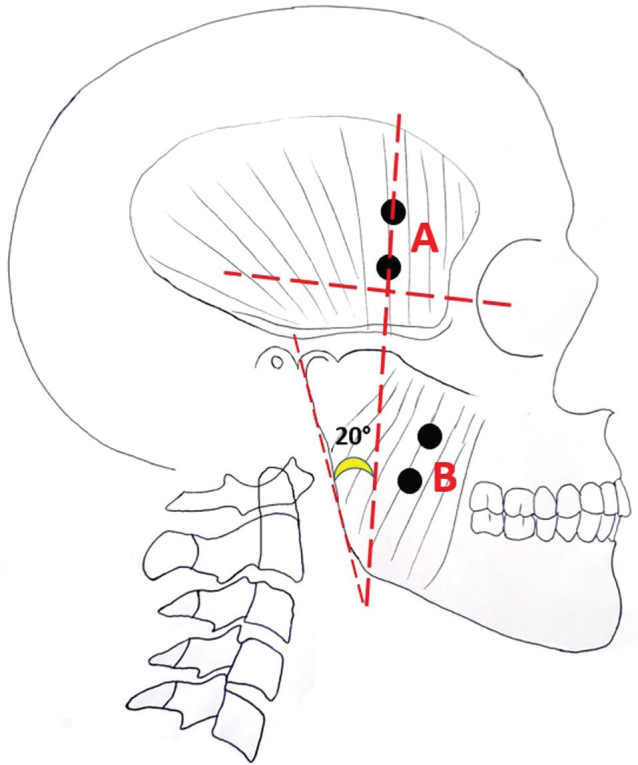
(A) Placement of electrodes on the anterior temporal muscle. (B) Placement of electrodes on the masseter muscle.

The placement of the electrodes on the recording areas was done using palpation. The patient was first asked to clench their teeth. The superficial belly of the masseter muscle was palpated near the gonion on the gonion-lateral canthus line. The anterior temporal muscle fibres were palpated along a line that makes a 20° angle with the ramus line (the line connecting the gonion to the condylar head) and extends laterally above the external ear. The electrodes were placed in alignment with the fibres of the masseter and anterior temporal muscles [36, 38] (Figure 3). All recordings were taken while the patient was in a seated position at rest, and the patient was instructed not to move any part of their body other than the desired jaw movements during recording.

In our study, a total of four EMG recordings were obtained for each patient during closure, swallowing, chewing, and clenching functions for the right and left masseter and anterior temporal muscles. The instructions given to the patient and the functional movements performed were as follows:

1. Closure: The patient was asked to close their teeth to make contact.
2. Clenching: The patient was asked to maximally and voluntarily clench their teeth while in contact.
3. Chewing: The patient was asked to chew two hazelnuts as they normally would.
4. Swallowing: The patient was asked to swallow the bolus after chewing as they normally would.

**Figure 4:**
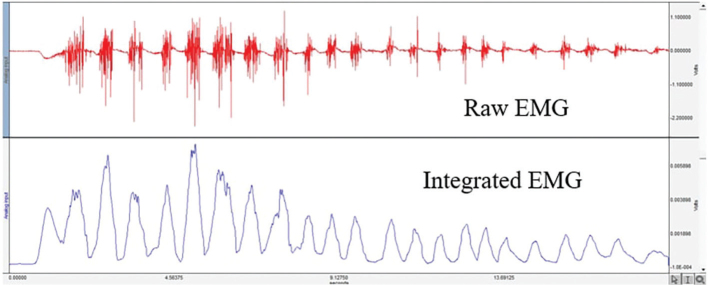
Display of raw and integrated electromyographic recordings.

All EMG measurements and evaluations were performed by the same orthodontist. Electromyographic recordings were taken at a sampling rate of 200,000 samples per second. The GAİN (gain) setting on the amplifier was adjusted to 1,000, and each recording lasted approximately 10 s. In order to perform evaluations based on integrated EMG data, all raw EMG data initially obtained were rectified in full-wave mode using the ‘AcqKnowledge’ programme (Figure 4). Then, the smoothing factor was set to 30 for all recordings. To facilitate calculations in the evaluations, all data were converted from volts (V) to millivolts (mV). For statistical evaluation, the average EMG values during the closing activity were taken, while the maximum EMG values were used for the clenching, chewing, and swallowing activities [12, 33, 39].

Analyses were performed using the ‘IBM SPSS 20’ (Statistical Packages for Social Sciences) (IBM Corp. IL, Chicago, USA) statistical analysis software. For each parameter examined, descriptive statistical values (mean, standard deviation, median, minimum, maximum) were determined. The normality of continuous variables was assessed using the Shapiro-Wilk test. As a result of this analysis, it was determined that the measurements used in our study did not show a normal distribution, so non-parametric tests were preferred for all statistical evaluations. The Friedman test was used to examine changes occurring between different recording periods. For parameters showing significant differences between recording periods, the Friedman two-way analysis of variance (ANOVA) by ranks (k samples) test was used as a post-hoc test. The level of statistical significance was set at *p* < 0.05.

## Results

In our study, EMG recordings were obtained from 26 patients (12 male, 14 female) with an average age of 21.7 years (ranging from 18 to 34 years) for the EMG assessments (Table 1). The follow-up period for each patient is 6 months, and the total duration of the study is 22 months.

**Table 1:** Demographic data.

Sex	Mean ± Standard deviation (age)	Sample size
Male	21.30 ± 2.311	12
Female	22.17 ± 3.096	14
Total	21.76 ±2.745	26

No statistically significant difference in activity was observed between periods during the closure and swallowing functions of the right masseter muscle (*p* > 0.05). Regarding the activity of the right masseter muscle during clenching, statistically significant differences were found between the T1–T0 (*p* < 0.001) and T2–T1 (*p* < 0.01) periods. In addition, for the activity during chewing, a statistically significant difference was found between the T1–T0 periods (*p* < 0.05) (Table 2).

**Table 2:** Statistical analysis results comparing changes in electromyographic activity (in millivolts) of the masseter muscle (right/left) between recording periods.

	T0		T1		T2		test value	*p*	Post hoc
	Mean ± SD	Median (min-max)	Mean ± SD	Median (min-max)	Mean ± SD	Median (min-max)			
Right Masseter Closure (mV)	0.26 ± 0.18	0.21 (0.1–0.83)	0.28 ± 0.2	0.23 (0–0.91)	0.28 ± 0.19	0.23 (0–0.73)	0.538	0.764 Σ	
Right Masseter Clenching (mV)	3.15 ± 1.96	2.57 (0.49–9.29)	1.84 ± 1.14	1.65 (0.21–4.46)	2.61 ± 1.83	2.35 (0.37–8.57)	15.077	0.001 Σ ***	T2–T1 (0.007) α** T1–T0 (0.001) α ***
Right Masseter Chewing (mV)	3.57 ± 1.95	2.98 (0.93–9.42)	2.35 ± 1.15	1.96 (0.83–5.88)	3.17 ± 1.84	2.57 (0.94–7.47)	8.524	0.014 Σ ***	T1–T0 (0.013) α *
Right Masseter Swallowing (mV)	1.71 ± 1.29	1.45 (0.32–5.30)	1.03 ± 0.47	0.96 (0.39–1.91)	1.21 ± 0.84	0.99 (0.37–4.41)	3.059	0.217 Σ	
Left Masseter Closure (mV)	0.32 ± 0.23	0.31 (0.1–1.29)	0.27 ± 0.18	0.22 (0–0.79)	0.25 ± 0.14	0.24 (0–0.77)	2.660	0.264 Σ	
Left Masseter Clenching (mV)	3.07 ± 1.55	2.96 (0.46–7.40)	1.88 ± 1.20	1.73 (0.20–4.84)	2.60 ± 1.88	2.25 (0.51–7.35)	16.231	0.000 Σ ***	T1–T0 (<0.001) α ***
Left Masseter Chewing (mV)	3.82 ± 2.25	3.52 (1.10–8.39)	2.39 ± 1.34	2.27 (0.83–8.06)	3.18 ± 2.06	2.58 (0.88–8.75)	11.308	0.004 Σ **	T1–T0 (0.003) α **
Left Masseter Swallowing (mV)	1.39 ± 1.06	1.19 (0.29–4.83)	0.96 ± 0.52	0.80 (0.36–2.67)	0.85 ± 0.48	0.72 (0.39–2.39)	3.769	0.152 Σ	

Σ: Friedman Test α: Friedman two-way ANOVA by ranks (k samples)

* *p* < 0.05

** *p* < 0.01

*** *p* < 0.001

No statistically significant difference in activity was found between periods during the closure and swallowing functions of the left masseter muscle (*p* > 0.05). However, for the activity of the left masseter muscle during clenching, a statistically significant difference was observed in the T1–T0 period (*p* < 0.001), and for the activity during chewing, a statistically significant difference was found in the T1–T0 period (*p* < 0.01) (Table 2).

There was no statistically significant activity difference between periods for the right anterior temporal muscle during the closure function (*p* > 0.05). For the activity of the right anterior temporal muscle during the clenching function, significant differences were observed between the T1–T0 (*p* < 0.001) and T2–T1 (*p* < 0.05) periods. During the chewing function, significant differences were noted between the T1–T0 (*p* < 0.001) and T2–T0 (*p* < 0.05) periods. In addition, for the swallowing function, significant differences were found between the T1–T0 (*p* < 0.05) and T2–T0 (*p* < 0.05) periods (Table 3).

**Table 3:** Statistical analysis results comparing changes in electromyographic activity (in millivolts) of the anterior temporal muscle (right/left) between recording periods.

	T0		T1		T2		test value	*p*	Post hoc
	Mean ± SD	Median (min-max)	Mean ± SD	Median (min-max)	Mean ± SD	Median (min-max)			
Right Anterior Temporal Closure (mV)	0.30 ± 0.17	0.26 (0.11–0.89)	0.27 ± 0.17	0.25 (0–0.90)	0.32 ± 0.20	0.24 (0.14–0.93)	0.835	0.659 Σ	
Right Anterior Temporal Clenching (mV)	2.30 ± 1.33	2.05 (0.60–6.86)	1.43 ± 0.74	1.33 (0.29–3.14)	1.67 ± 0.66	1.67 (0.44–3.46)	15.864	0.000 Σ ***	T2–T1 (0.013) α * T1–T0 (<0.001) α ***
Right Anterior Temporal Chewing (mV)	2.30 ± 1.41	1.95 (0.77–7.95)	1.37 ± 0.57	1.37 (0.40–3.10)	1.56 ± 0.79	1.33 (0.69–4.25)	15.864	0.000 Σ ***	T1–T0 (<0.001) α *** T2–T0 (0.013) α *
Right Anterior Temporal Swallowing (mV)	1.17 ± 0.76	0.95 (0.29–2.86)	0.68 ± 0.37	0.60 (0.17–2.04)	0.60 ± 0.32	0.48 (0.21–1.58)	9.922	0.007 Σ **	T1–T0 (0.031) α * T2–T0 (0.013) α *
Left Anterior Temporal Closure (mV)	0.32 ± 0.25	0.25 (0–1.34)	0.33 ± 0.19	0.29 (0.10–0.86)	0.28 ± 0.12	0.27 (0.15-0.66)	0.608	0.738 Σ	
Left Anterior Temporal Clenching (mV)	2.10 ± 1.11	2.0 (0.76–6.78)	1.45 ± 0.78	1.14 (0.30–3.01)	1.69 ± 0.93	1.33 (0.43–3.68)	17.538	0.000 Σ ***	T2–T1 (0.038) α * T1–T0 (<0.001) α ***
Left Anterior Temporal Chewing (mV)	1.97 ± 0.98	1.78 (0.60–5.09)	1.45 ± 0.57	1.37 (0.60–2.76)	1.65 ± 0.75	1.59 (0.61–3.36)	13.231	0.001 Σ ***	T1–T0 (0.001) α ***
Left Anterior Temporal Swallowing (mV)	1.08 ± 0.61	1.00 (0.25–2.56)	0.80 ± 0.40	0.68 (0.35–2.09)	0.70 ± 0.55	0.58 (0.25–2.96)	13.767	0.001 Σ ***	T2–T0 (0.001) α ***

Σ: Friedman Test α: Friedman two-way ANOVA by ranks (k samples)

* *p* < 0.05

** *p* < 0.01

*** *p* < 0.001

Similarly, the analysis revealed that there was no statistically significant activity difference between periods for the left anterior temporal muscle during the closure function (*p* > 0.05). For the activity of the left anterior temporal muscle during the clenching function, significant differences were found between the T1–T0 (*p* < 0.001) and T2–T1 (*p* < 0.05) periods. During the chewing function, significant differences were observed between the T1–T0 (*p* < 0.001) periods. Lastly, for the swallowing function, significant differences were noted between the T2–T0 (*p* < 0.001) periods (Table 3).

## Discussion

According to the findings of this study, partial changes in the EMG activity of the masticatory muscles for certain functions were observed within the third month. However, no significant overall change was recorded in the 6-month follow-up evaluation. These results are consistent with our null hypothesis (H₀): ‘Bimaxillary orthognathic surgery does not cause a significant change in the EMG activity of the masticatory muscles’. Therefore, although some temporary changes were detected, the general EMG activity of the muscles remained stable over a 6-month period, which is considered a short time in terms of orthodontic and orthognathic treatments, supporting the null hypothesis (H₀).

There are numerous studies evaluating the effects of different skeletal malocclusions on masticatory muscles from an EMG perspective [40–45]. These studies have demonstrated significant differences in the EMG activities of the muscles between individuals with malocclusion and healthy individuals [40–44].

Changes in masticatory muscles following orthognathic surgery have been the subject of numerous studies, examining alterations in muscle volume, activity, dimensions (thickness and width), elasticity, muscle fibre orientation, and bite force. These changes have been investigated using methods such as electromyography, ultrasonography, ultrasound elastography, computed tomography, magnetic resonance imaging, and pressure-sensitive bite paper [29, 46–49]. Among these methods, electromyography is the most reliable technique, as it objectively reveals the actual state of a muscle during various movements, as well as muscle interactions and coordination, compared to other methods [8, 50]. All these studies indicate that significant structural and functional changes may occur in muscles after orthognathic surgery, and these changes can be identified by evaluating the EMG activity of the muscles. Therefore, in our study, we aimed to assess the changes in masticatory muscle activity using EMG following bimaxillary orthognathic surgery in individuals with skeletal Class III malocclusion.

In the majority of studies investigating changes in EMG activity of masticatory muscles following orthognathic surgery in individuals with skeletal Class III malocclusion, the masseter and anterior temporal muscles have been primarily evaluated [20–22, 24, 25, 30, 31, 34, 51–53]. Among the key reasons for evaluating these muscles are their active role in jaw functions and the ease of determining their localisation [5, 38, 54].

In the literature, studies evaluating changes in muscle EMG activity following orthognathic surgery have reported varying findings. Some studies have indicated that muscle activity decreases in the early postoperative period and then increases [21], while others have reported an increase in muscle activity [21, 24, 25, 27, 29, 34, 53] or no significant change [20, 23]. These findings suggest that the EMG activity of muscles may vary depending on the type of surgery performed and the timing of the evaluation.

Çelakıl et al. investigated changes in the EMG activity of the anterior temporalis and masseter muscles during rest and clenching functions in skeletal Class III malocclusion patients who underwent double-jaw surgery. Measurements were taken immediately after splint removal and at 1 month and 6–8 months postoperatively. The researchers reported a significant decrease in EMG activity of the anterior temporalis muscle at 1 month postoperatively, whereas no significant change was observed in the masseter muscle at this time. At 6–8 months postoperatively, the resting EMG activity of both muscles showed a significant decrease, reaching values similar to the control group. Meanwhile, a significant increase in EMG activity was observed during functional movements; however, despite this increase, the values remained lower than those of the control group [21].

Nakata et al. evaluated changes in the EMG activity of the temporalis and masseter muscles during clenching and chewing functions in patients with mandibular prognathism who underwent mandibular setback surgery. Assessments were conducted at the end of orthodontic treatment, and 2 years and 7 months postoperatively. Contrary to the findings of our study, the researchers reported an increase in the activity of these muscles after surgery. However, they noted that this increase was not at the desired level and that muscle activity remained lower than that of the control group postoperatively [25].

We attribute the difference between our findings and those of Nakata et al. and Çelakıl et al. to the discrepancy in measurement time points.

Ko et al. investigated the EMG activity changes in the anterior temporalis and masseter muscles during resting and clenching functions at 1 and 6 months after bimaxillary orthognathic surgery in patients with skeletal Class III anomalies [23]. These researchers reported that, during all functions and at all recording periods, the activity of the masseter muscle was lower than that of the anterior temporalis muscle. They also found that there were no significant long-term changes in either muscle during the resting function. In addition, they observed that EMG activity during the clenching function significantly decreased in both muscles at 1 month, followed by an increase approaching baseline values. As of the 6-month measurements, our findings are consistent with theirs.

Muftuoglu et al. evaluated the changes in the EMG activity of the masseter muscle in patients with skeletal Class III malocclusion who underwent bimaxillary orthognathic surgery, at 3 months and 1 year post-surgery [29]. The researchers reported that the EMG activity of the masseter muscle during maximum clenching was significantly higher in the control group across all recording periods, and that there were significant increases in the masseter muscle’s EMG activity at both the 3-month and 1-year marks in the surgical group. In the study group of Muftuoglu et al., in addition to patients who underwent double jaw surgery, there were also patients who had only mandibular setback surgery.

Kobayashi et al. evaluated the changes in the EMG activity of the masseter and temporal muscles in patients with mandibular prognathism who underwent orthognathic surgery, comparing them with a control group at 6 months, 1 year, and 2 years post-surgery [24]. The researchers reported that the masseter and temporal muscle activities increased after the surgery, but in the long term, the activity of these muscles remained significantly lower than the control group. Only a small portion of the study group in Kobayashi et al.’s research had undergone bimaxillary orthognathic surgery.

We believe that the differences between our findings and those of Muftuoglu et al. and Kobayashi et al. are due not only to the different measurement times, but also to the variation in the surgical procedures within the study groups.

Farronato et al. assessed the EMG activities of the anterior temporal and masseter muscles during maximum clenching function in patients with skeletal class II and III anomalies who underwent orthognathic surgery [34]. The researchers reported that in the class III group, there was an increase in all muscle activities during maximum clenching function at the end of treatment compared to preoperative levels. Our findings are not consistent with those of Farronato et al. Because of the lack of clear information regarding the type of surgery performed and the evaluation times in their study, it has not been possible to fully explain the discrepancies between their findings and ours.

Trawitzki et al. evaluated changes in the EMG activity of the masseter and temporal muscles during maximum tooth clenching and chewing in patients with skeletal class III anomalies who underwent orthognathic surgery, 6–9 months post-surgery [27]. They reported a significant increase in the EMG activity of the temporal and masseter muscles after the surgery, but even at the end of this period, the activity levels remained lower than the control group. In another study, Trawitzki et al. examined the EMG activity of the temporal and masseter muscles during chewing and clenching function 3 years after orthognathic surgery in a similar patient group [53]. They found significant increases in EMG activity of the chewing muscles compared to preoperative levels, with masseter muscle activity showing values closer to the control group. We believe that the differences between their findings and ours may be influenced by the fact that Trawitzki et al. performed bimaxillary orthognathic surgery on only a very limited number of patients, conducted evaluations 3 years after the surgery, and applied myotherapy to the patients.

One of the limitations of our study is the inability to investigate changes that may occur over long periods, which are considered extended durations in orthodontics, due to time constraints. In addition, EMG recordings could not be obtained on the same day and time of the week for each patient. The primary reason for conducting the first postoperative evaluation at 3 months was that many patients had less stable occlusion in the first 1–2 months compared to the third month, and some patients were still experiencing postoperative pain or using analgesics. For these reasons, we considered data collection at the third postoperative month to be more reliable. However, the absence of first-month measurements could be considered a limitation, as a sufficient number of patients might have had stable occlusion at their 1-month follow-up, and some of them might not have experienced pain or the need for analgesics.

Another limitation of our study is that the results were not interpreted using specific indices (such as asymmetry and torque indices). If evaluations had been performed using indices – for instance, the asymmetry index – we could have assessed the relative activities of opposing muscles. In addition, when forming patient groups, the degree of mandibular asymmetry could have been taken into account. However, given the limited time available for this study, there was a high risk of not achieving sufficient sample sizes within the groups.

Furthermore, based on our literature review, we could not find similar studies that considered gender differences. In our study, as in others, the groups were not divided based on gender, which could be seen as a general limitation. If gender-based groupings had been performed, the clinical and academic value of the study might have been somewhat enhanced. Lastly, another limitation is the absence of a patient group undergoing myofunctional therapy. If such a group had been included, the clinical and academic value of the study could have increased. Of course, many of these limitations stem from the study’s time constraints; otherwise, they could have been addressed.

To summarise the findings of our study, there was no significant change in the EMG activity of the masticatory muscles by the sixth month after surgery. Clinically, the fact that the EMG activity remained more stable, rather than showing significant increases or decreases, can be considered a positive indicator for the potential relapse risk in these patients. In addition, the overall stability observed during the 6-month period questions the necessity of myotherapy interventions during the postoperative rehabilitation process. From an academic perspective, our study is one that evaluates the EMG activity of masticatory muscles during functions such as clenching, biting, chewing, and swallowing in patients undergoing orthognathic surgery. Specifically, testing chewing function with a real food item, such as a nut, makes a significant contribution to methodological diversity in this field. Future studies could explore the sustainability of these findings with longer follow-up periods.

## Conclusion

No significant change was observed in the EMG activity of the masticatory muscles during the 6-month period following bimaxillary orthognathic surgery. Since our study covered a 6-month postoperative period, further studies are needed to address the question of how EMG data, which examine changes after bimaxillary orthognathic surgery, would change over a longer period. These studies should involve larger and more diverse sample groups and evaluate changes occurring over extended periods.
